# Desert Dust and Health: A Central Asian Review and Steppe Case Study

**DOI:** 10.3390/ijerph14111342

**Published:** 2017-11-03

**Authors:** Troy Sternberg, Mona Edwards

**Affiliations:** 1 School of Geography, University of Oxford, South Parks Road, Oxford OX1 3QY, UK; 2 Oxford Rock Breakdown Lab, School of Geography, University of Oxford, South Parks Road, Oxford OX1 3QY, UK; mona.edwards@ouce.ox.ac.uk

**Keywords:** dust, health risk, particulates, Central and Inner Asia, Mongolia, desert, mining, exposure

## Abstract

In Asian deserts environmental and anthropomorphic dust is a significant health risk to rural populations. Natural sources in dry landscapes are exacerbated by human activities that increase the vulnerability to dust and dust-borne disease vectors. Today in Central and Inner Asian drylands, agriculture, mining, and rapid development contribute to dust generation and community exposure. Thorough review of limited dust investigation in the region implies but does not quantify health risks. Anthropogenic sources, such as the drying of the Aral Sea, highlight the shifting dust dynamics across the Central EurAsian steppe. In the Gobi Desert, our case study in Khanbogd, Mongolia addressed large-scale mining’s potential dust risk to the health of the local population. Dust traps showed variable exposure to particulates among herder households and town residents; dust density distribution indicated that sources beyond the mine need to be considered when identifying particulate sources. Research suggests that atmospheric dust from multiple causes may enhance human particulate exposure. Greater awareness of dust in greater Central Asia reflects community concern about related health implications. Future human well-being in the region will require more thorough information on dust emissions in the changing environment.

## 1. Introduction

In Asian deserts environmental and anthropomorphic dust is a significant health risk to populations [1]. Natural particulate sources in dry landscapes are exacerbated by human activities that increase the vulnerability to dust and dust-borne disease vectors. Today in Central and Inner Asian steppe drylands, agriculture, mining, and rapid development contribute to increased dust generation and community exposure. Perhaps most infamous is the desiccation of the Aral Sea into a major dust source, which the UN called one of the world’s ‘worst environmental disasters’ [2]. This highlights that ‘Asia sources, including the Taklimakan, Gobi, and the Chinese loess plateau represent ~25% of global dust emissions’ ([3], p. 1). Whilst dust impact on regional health has been identified but not studied extensively [4,5,6], knowledge of potential desert dust interaction with human health is well established [7]. This paper examines the current dust-health dynamics across greater Central Asia, and then investigates a case study in the Mongolian Gobi to examine if mega-mining dust generation may present a particulate concentration sufficient to affect human health.

Dryland dust storms and particulate matter are significant globally because they affect the physical and human environment, both in situ and in downwind locations [1]. Arid zones are identified as major dust sources with Asian hotspots, such as the Aral Sea, Taklamakan, and Gobi Deserts documented as home to significant dust events [4,8,9]. The importance of Central and Inner Asia as a source is reflected in regional dust affecting Japan, Korea, and Taiwan [10] with recent investigation showing that 3% of Asian dust now reaches the western United States (US) [3]. Climate variability, land cover change—especially mining, agriculture and development—and natural source exposure contributes to the Asian drylands’ role as a significant dust source.

**Figure 1:**
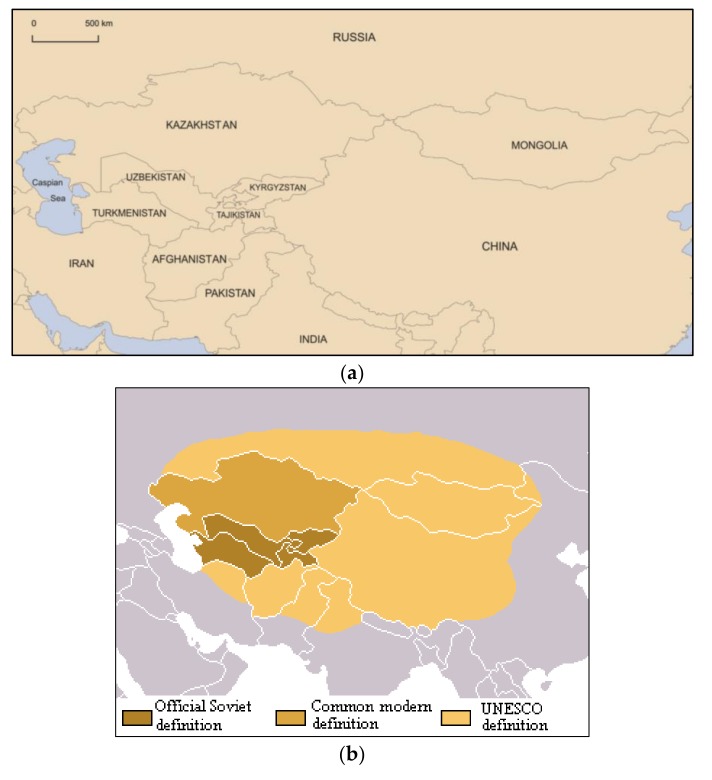
Defining Central Asia—(**a**) identifies national borders in the region; (**b**) reflects Soviet, common and UNESCO definitions of Central Asia covering vastly different regions [16].

The greater Central Asian region represents inland, continental, high latitude cold deserts (Figure 1a) that comprise a part of the vast Asian paleo-arctic arid expanse. Known under several loosely defined terms, Mohammat et al. [11] identify Inner Asia as stretching from Mongolia to the Himalayas and the Caspian Sea (35–55° N, 45–120° E). Lioubimtseva and Henebry [12] delineate the political construct of Central Asia as based on Soviet-era borders—today’s Kazakhstan, Kyrgyzstan, Tajikistan, Turkmenistan, and Uzbekistan. Various definitions include parts of Afghanistan, Tibet, and even Iran or southern Russia (Figure 1b) in a more geographic demarcation. In Russian, the term Middle Asia remains standard [13], whilst Central Eurasia, High Asia, Asian Interior, and historically Turkestan have named the region [14]. This paper defines Central Asia and Inner Asia to include the areas covered above and presented in Figure 1. The vast region, covering >5 million km^2^ and being home to ~100 million people, encompasses several deserts and vast steppe drylands that serve as extensive global dust emission sources [8]. This paper presents the regional dust context through assessment of recent dust research in the area; it then examines how dust impacts human health and notes implications of mining-generated particulates. A case study is then presented in the Mongolia Gobi Desert (43.20° N, 107.19° E), where an in-situ investigation was conducted through placement of dust traps to assess the potential nexus between mining-dust-health in the rural Central Asian environment. Work investigates dust in the vicinity of an international mega-mine in Mongolia, targeted by a community complaint to international lenders [15] and the potential community health exposure to dust, a risk that repeats throughout Central Asia.

### 1.1. Regional Context: Description of Dust Sources 

Dust storms are meteorological events that are observed globally, often in arid and semi-arid climates and are common in the dust belt that stretches from the Sahara in West Africa to the Taklamakan and Gobi deserts in East Asia [17]. In Central Asia, the main dust sources includes (i) the Taklamakan desert [18] of the Tarim Basin [19] in the Northwest of China; (ii) the Badan Jarain desert [20], which spans the provinces of Gansu, Ningxia, and Inner Mongolia of China; (iii) the Gobi desert with its Altai Mountains, Lake Ulaan-Nuur, Zamiin-Uud of Mongolia; (iv) areas along the Hexi corridor in China; (v) the Karakum of Turkmenistan; (vi) Kyzylkum of Uzbekistan; and, (vii) the Arakum and the Balkhash-Alakol depression of Kazakhstan [21].

Dust emissions occur where strong wind that exceeds a threshold value, surface material is susceptible to wind erosion and transport, there is limited vegetation cover and instable atmospheric conditions [21]. Estimations of dust emissions strength [22,23] in different parts of the world demonstrated that after the Sahara’s importance, Central Asia accounts for 20% of the global total [1]. Kes et al. [19] reported that the Tarim Basin in North-West of China, with 100–174 dust storms per year, has more dust events than anywhere on Earth. In general, most regional dust storms last between 2 to 21 h [24], however fine PM_10_ and PM_2.5_ remain in the air [1]. These factors are a product of wind speed, visibility, event duration, climate modification, and a new meteorological phenomenon-radiation budget. This region, far from any oceans, has about 100–400 mm of precipitation and up to 900–1500 mm of evaporation per year [25]. Consequently, the region is characterised by vast desert areas, frequent soil, and atmospheric droughts [21] and strong near surface winds. This Central Asian Arid Zone (CAAZ) is a powerful source of airborne dust that has a major impact on various earth ecosystems [26].

Asian dust constitutes an important component of mineral aerosol affecting the global water cycle and energy budget [27]. The weathering and dissolution of Asian dust absorb CO_2_, as well as other vital nutrients that are transported to remote terrestrial and marine ecosystems, making it a part of global elemental cycling [28] and affecting the paleo-environmental data [29,30,31]. Zheng et al. [32] state that Asian dust includes almost all mineral types that are part of the upper continental crust and that, due to wind sorting; it is also mainly composed of light minerals and clays mineral. Soils normally have specific clay mineral composition that is equilibrated with the local climate condition; for example, Biscaye et al. [33] first used the kaolinite/chlorite ratio of clay minerals to detect the provenance of dust. Then Shen et al. [34] extended this method to illite/kaolinite ratio as well to investigate the source of the suspended particles in North China. Unfortunately, this method was not very successful for the Asian dust to detect difference of clay mineral composition between the possible source areas for the provenance. When coupled with heavy metals composition, magnetic susceptibility, X-ray diffraction was tried but was not a reliable source tracer for Asian dust. Some positive detection methods were obtained with carbonate contents analysis, especially dolomite [28], and with silicate Nd-Sr isotopic composition [35,36,37]. These methods effectively analyse the geo-chemistry of dust in Asia.

Past studies have indicated that many dust emissions are consistently from active hot spots. Three main factors are the contributors of the dust activity. The first factor is natural climatic variability. For example, links have been established between dust emissions from the Tarim basin and the Artic Oscillation (AO) Index, with dust activity being high during the negative phase of the AO [18]. Anthropogenic modifications of the desert surface [38] and global warming climate changes are the two other contributing factors of the dust activities [1]. Central Asia is probably the most disturbed desert surface with grazing and crop production, desiccation of lakes and soil surfaces by inter-basin water transfers, vehicular traffic, ground water depletion, and the removal of vegetation cover [21].

Some source areas are very active dust generators; in Central Asia, the Tarim Basin/Taklamakan [18,19], the Badan Jarain [39], and the Gobi of Mongolia [40] are important sources of dust storms (Table 1). Dust exposition can also come from anthropogenic sources, such as industrial, agricultural, and mining exploitations that generate higher concentration levels of a given substance in the air [1]. The Central Asian landscape has been altered, in part being degraded by extensive farming programmes and mining extraction [4]. The dust impact on human health can occur at great distance from the source, dependent on particulate transport [41].

Dust storms in Central Asia have been studied and recorded since 1936, mainly before 1980 [42]. Being a major source of global dust aerosol, Central Asia has been a generator of frequent and severe dust storms [13]. Analysis of meteorological data establishes changes in dust events and frequency. Declining trends were observed during the latter part of the 20th century (1970–2000) in Turkmenistan [43] and Central Asia [21], as well as in parts of Mongolia and China [44,45] during the five last decades of the 20th century. In contrast, others found an increasing trend reflecting changes in precipitation and soil moisture, rather than wind conditions [46]. Goudie [1] identified that natural and anthropogenic factors are implicated in the observed trends [47]. The early years of the present millennium saw several severe dust events in this region [48,49]; the prevalent explanation is that natural climatic factors are surpassing the human pressures on the land [50].

**Figure 2:**
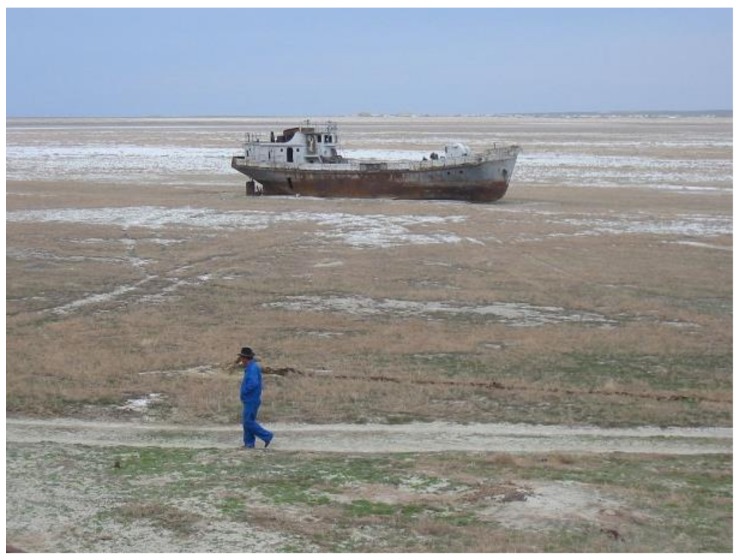
Ships abandoned in desiccated parts of the Aral Sea [55].

During the Soviet era, Central Asia experienced severe land degradation with excessive livestock exploitation and radical transformation of agricultural practices [13]. In Kazakhstan, Turkmenistan and Uzbekistan land degradation followed Khrushchev’s infamous, environmentally damaging, ‘Virgin lands’ farming expansion diktat [51]. Their natural desert pastures have been transformed into vast agricultural exploitations [4,52]. Against the warning of scientists, Soviet management turned out to be extremely inefficient despite vast irrigation expansion programmes. Due to its overexploitation as water resource for agriculture, the Aral Sea basin lost more than 70% of its volume in five decades and the region has been proclaimed an ecological disaster zone [4,53]. Water demand from the Amu Darya and Syr Darya rivers, as well as the Kara Kum Canal of Turkmenistan kept increasing, which resulted in Aral Sea water table collapse [26]. As a consequence of the drop in the Aral Sea level, new dry areas became active hotspots of dust storms [4]. The bottom of the sea salts, and then fertilisers, pesticides, herbicides, and other conditions that affect health and hygiene (especially water and sewage), supplied the dust storms with particles that are chemically detrimental to human health. The Aral Sea loss [54] is a striking example of how disturbance of the natural landscape contributes to dust and health impacts. Intensive water diversion upriver for farming has exposed the seafloor and salty flats that are now the source of severe dust storms with very high amount of PM_10_ particulate (Figure 2) [5].

**Figure 3:**
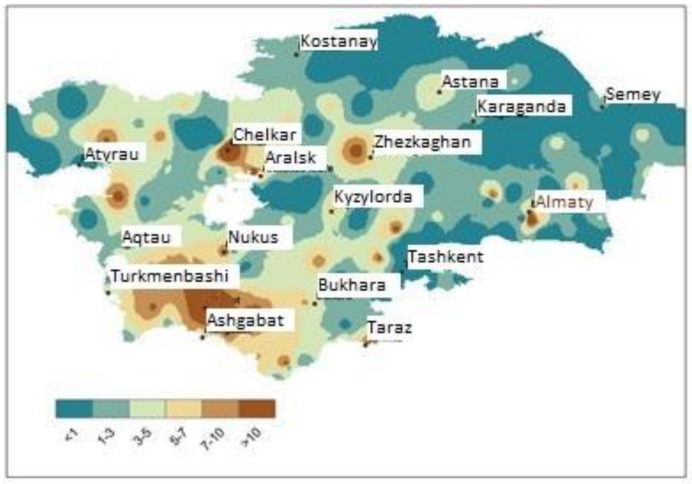
Spatial distribution of severe and very severe dust storms in Middle Asia, 2013. Darker colour represents higher intensity [13]. Note: stress is on severity rather than town name.

Indoitu et al. [21] investigated dust storms in Middle Asia (their term), describing seven decades of dust storm phenomenon that cover the pre- and post-Soviet period (see also [13,42]). Research concludes that (i) generally the northern region has less frequent and shorter dust storm events; (ii) the southern region has higher frequency and longer dust storms; (iii) the spatial distribution analysis of dust storms revealed sources that have undergone changes during the time of analysis; (iv) the northern Caspian desert dust storm emission areas reduced significantly in size and shifted to the east; (v) in the Kara-kum and Kyzyl-kum deserts and the Balkhash Lake area dust storm emission areas reduced in size; and, (vi) the new Aral-kum human-induced desert, which was once a seafloor became very active [21]. The severe dust storms classified as hazardous and highly hazardous coincided with those with a duration that exceeded 20 days and were located in the north-western part of the Ili valley and the Kara-kum and Kyzyl-kum deserts (Figure 3).

Study of northern China and Mongolia from MODIS satellite images [56] enabled the identification of dust emissions hot spots. Dust sources in south-eastern Mongolia have migrated northward since 2006; dry lakes, riverbeds, mines, and croplands contribute as hot spots in China and Mongolia. Industrial activities in Otintag Sandy and agricultural activities in Horquin sandy land are identified hot spots on MODIS [56]. Natsagdorj, L. et al. [40] used climatology data from 1937 to 1999 to analyse dust storm events in Mongolia, thereby confirming the Gobi as a major source of dust storms. The highest frequency of dust storms is observed in the southern Altai Mountains, around Lake Ulaan-Nuur and Zamiin-Uud on the border. Findings coincide quite well with the strong wind events frequency [57]. First, dusty days have increased from the 1960s to the 1990s, and then started to decrease from 1990, confirming [44] findings. Results showed that 61% of the dust storms occur in the spring, which concurs with the well-known Spring Asian Dust Storms (SADS) in Japan [58].

### 1.2. General Assessment of Dust-Health Interaction with a Focus on Mongolia

Asian dust, produced by the land surface dominated by gravel deserts, sandy deserts, sandy land, and dry grassland is ideal for dust emission [59]. The released dust transports eastward and south-eastward with the atmospheric circulations is known as spring dust storms in Central and East Asia. Dust matter is absorbed during transport influences the atmospheric quality of the populated regions [28]. Natural aeolian dust and sand can be beneficial; agricultural loess deposits are aeolian sediments that are formed by the accumulation of wind-blown dust, are common on the northern China plateau [60]. This is exacerbated when dust emission intensity and impacts are amplified by anthropogenic interventions that it becomes a source of air pollution [61,62]. Its constituent parts are airborne particulate matter (PM) from any direct emissions, which could have been modified with secondary products emitted from anthropogenic activities or biogenic origins. 

Level and exposition rate: In the Inner Asia region, home to 20% of global dust, health risk varies with the exposure, frequency, and intensity of dust storms [1]. Analysis of meteorological data establishes changes in dust events and frequency. Declining trends were observed during the latter part of the 20th century (1970–2000) in Turkmenistan [43] and Central Asia [21]. In general, most regional dust storms last between 2 to 21 h [24], however fine PM_10_ and PM_2.5_ remain in the air [1]. These factors are a product of wind speed, visibility, event duration, climate modification, and new meteorological phenomenon-radiation budget.

Studies emphasise the possible harmful effects of high concentrations of airborne PM on human health with a focus on how particulate affects cardiovascular and respiratory functions [63]. Scientific evidence identifies the potential effects of PM_10_ and PM_2.5_ on our body systems; pathogenic effects at different levels can appear at different time scales with a range of plausible disorder symptoms. Epidemiological studies indicate the possible health impacts of dust exposure, yet direct causality is difficult to document. For example, Kenessariey et al. [64] examined the human health cost of air pollution in Kazakhstan and the direct effect of high levels of PM_10_ and PM_2.5_ in the air on the mortality rate. Added to the high concentrations in small PM, the ambient air in large urban, industrial, and agricultural areas of Central Asia is polluted by chemical emissions. The systemic review in Mongolia done by Jadambaa et al. [65] shows the extent of the environmental pollutants and risk factors in rural and urban areas in Mongolia. Results demonstrated that Mongolian children have greater exposure to environmental factors with polluted air that includes heavy metals and tobacco smoke.

The human body is capable of auto-cleaning and auto-healing through specialised cells whose functions are to sweep or phagocytised away dust out of our body and organs that eliminate pathogenic agents [66,67]. Unfortunately, in several areas of Inner Asia environmental risks factors now exceed the human capacity to mitigate damage [68]. The severity of dust health impacts depends on individual tolerance levels, which is linked to genetic background, age, and circumstances affecting the ability to cope with the aggressive agents and levels of exposure to environmental risk factors.

Several determinants affect the aeolian dust effect on human health. Assessing the frequency and severity of the pathogenic effects encourages a better understanding of the possible different detrimental impacts of dust on the human body [1]. Particle size is a main determinant; unlike coarse PM that are filtered by the ciliated and mucus cells, PM < 10 microns in diameter are absorbed by the lung tissues and can embed in the bronchioles and alveoli structures [69]. This affects respiratory functions as fine grains (<2.5 microns) can enter the blood stream and are transported to other organs to act as disrupters [70,71]. Khaniabadi, Y.O. et al. [72] demonstrated that dust events and PM_10_ have a direct impact on hospital admissions for Chronic Obstructive Pulmonary Disease (COPD) and on the Respiratory Mortality (RM). 

The chemical composition of particulate—mineralogical, isotopic, and elemental—is an important factor. For example, Long-term exposure to silica dust is associated with an increase of mortality among the mineworkers due to respiratory diseases, silicosis, lung cancer, and cardiovascular diseases [73]. Generally speaking, dust storms are composed primarily of Silica SiO_2_, Al_2_O_3_, and in lesser amounts, Fe_2_O_3_, CaO and MgO. Other components in dust can be salts, organic, pathogenic, and polluting materials. Various studies examined the environmental risk factors due to SO_2_, NO_2_, Pb, and other heavy metals like Zn, Ni, Co, and Cr in the air near mining areas [74]. Geochemical analysis on Asian dusts indicated a clear contrast between the potential sources areas coming from the tectonic structure of the arid lands and gave some evidence of the natural or anthropogenic type of sources.

Anthropogenic sources, particularly mining, agriculture, industry, livestock grazing, and urban development are changing the dust composition and contaminants [4,75]. Among the transported anthropogenic pollutants, aerosols like pesticides, herbicides, and dioxins are well known for their effects on human health. For example, Kanatani, K.T. et al and Watanabe, M. et al. [76,77] found that the transport of pollen brought by the Asian dust Storms (ADS) increases asthma in the population and causes an increased morbidity in Japan [78]. Biological materials like bacteria, viruses, fungi, and pollen are also picked up and transported on long distances. Here, again human activities can modify the ecosystems, which will have a similar effect on dust composition.

Mining processes are a significant source of dust with exploration, development, extraction, and transport reconfiguring landscapes and contributing to emissions [79,80]. Nations across greater Central Asia host large-scale mines, excavating copper, gold, coal, uranium, silver, amongst several elements. The economic value, jobs and importance for state revenue leads to mining expansion despite possible detrimental environmental impacts. In Kyrgyzstan, gold and in Mongolia copper are the countries’ main exports, whilst China’s Gobi region is its largest coal producer and domestic energy source. Yet, mining generates copious dust, including finer particles that have respiratory health implications [79]. Open pit mines, rock crushing, waste heaps, and land cover change become dust sources. Ma et al. [74] illustrate how mining sites contaminate soil and dust, and contribute to air pollution after dust events [81] identify the high impact of mining on the environment and community and threatens herding livelihoods. In fact, ice cores in the Tien Shen Mountains identify recent dust deposition from Central Asian mining and anthropogenic causes [82]. As mining expands, the impact on dust generation will increase across the region.

There are predictive methods to assess the effects of dust pollution on human health based on observations and monitoring of the dust air composition and concentrations in different target pollutants, and using World Health Organisation (WHO) air qualities guidelines [83,84]. WHO estimations are based on available concentration data and evidences of the mortality effects of air particulate pollution [85]. The USEPA (United States Enivronmental Protection Agency) risk assessment model calculates of daily intake dose values using factors such as dust exposure frequency, duration, contact time, ingestion rate, inhalation rate, dermal adsorption factor, and the particle emission rate. Health risk models are used that are based on the carcinogenic or non-carcinogenic nature of the polluting substances being analysed [86]. Ghorbel, M. et al. [87] undertook health hazard assessment and calculation of airborne metals concentrations from the PM_10_ based on inhalable particle size. Ma et al. [74] and Li et al. [88] used the geo-accumulation index; Argyraki, A [89] and others introduced the notion of bioavailability and bioaccessibility to quantify the risks that are associated with exposure to environmental pollutants [90]; evaluations are based on in vivo animal experiments. Hospital admissions fore dust complications are a further method [76]. Studies in China link dust events to respiratory problems, such asthma, pneumonia, and tracheitis [67]. 

## 2. Materials and Methods

### 2.1. Mongolian Case Study: Context 

Mongolia has been called ‘the next Qatar’ for its great abundance of mineral resources and small population [91]. At the same time, it is considered as a major natural dust source [92]. In Southern Mongolia, two of the world’s largest mines—the vast Oyu Tolgoi copper/gold excavation and Tavan Tolgoi coking coal mine—have provided >US$1 billion in tax revenue at significant environmental and social costs. As Rio Tinto Corporation expands its $12 billion Oyu Tolgoi (OT) mine, there have been several concerns about dust-related health impacts of the mega-mine on local communities [80], a concern acknowledged by the mine [93]. Road dust generation (paved vs. unpaved), particulate implications for animal health and dust mitigation efforts by the mine have been significant citizen concerns. Conditions have resulted in formal complaints to the World Bank and attempts to halt mining for failing to address detrimental impacts [15,94]. Efforts in Mongolia reflect increased awareness of the potential damage caused by mining processes. New research in Mongolia is concerned with dust impact on both human and livestock health (Table 2). This paper goes a step further in investigating widespread dust in the vicinity of the Oyu Tolgoi mine.

Several impacts of mining and their related infrastructure and development have disrupted herder communities and environments, with potential severe implications human health and well-being. Foremost are the threats to health posed by dust and changes to water quality. This is a critical issue in Mongolia as nomadic pastoralism is the primary livelihood for 30% of the population [101], and provides the majority of the country’s food. Dependence on natural factors, including extreme cold, low moisture levels, and limited plant productivity, creates an unpredictable environment for both animals and herders [101]. Recently, activities at the two new mines have reconfigured this vast region of southern Mongolia. In this assessment, we focus on potential dust impact on the immediate community of the Oyu Tolgoi (OT) mine [100] to investigate the potential implications of mining dust generation on human health in the region. 

### 2.2. Study Site

**Figure 4:**
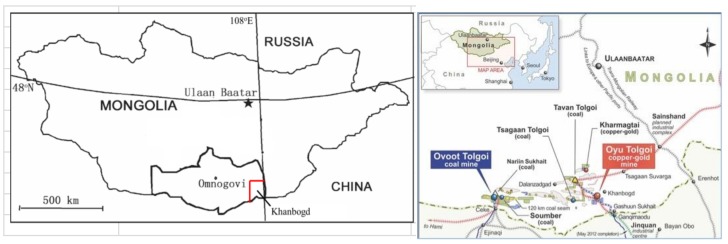
Map showing Khanbogd Soum, Mongolia (**left**); (**right**) mining sites, including Oyu Tolgoi, in Omnogovi Province, Mongolia [99].

South Gobi Province (Omnogovi) represents a high latitude, windswept desert environment with little built infrastructure (Figure 4). Herders live and animals graze near mine sites and use local groundwater as livestock provide a diet of meat and dairy; the shared landscape presents an ideal field study site to investigate human exposure to dust dynamics. For example, Rio Tinto’s copper extraction site occupies an 8 × 10 km exclusionary Mine License Area (MLA), where a huge open-pit mine generates copious dust from mineral digging, crushing, and transport. Bioavailability of dust, particulate composition, wind turbulence, and infrastructure fragmentation reconfigure compound dust exposure. Current research identifies the centrality of dust concern to area residents [15,80]; however, integrated examination and documentation does not exist. In this scenario, health risk becomes the focus between herders, the extractive industry, and governance in the region.

This study is focused on Khanbogd Soum. The Mongolian word Soum refers to both the administrative district (>13,000 km^2^) and the only town (population > 3000; 43.20° N, 107.19° E). The rolling arid landscape is situated ~900 to 1300 m a.s.l., with a prominent caldera in the Soum centre providing the name. Khanbogd is the warmest Soum in the country (average annual temperature 7–8 °C), and is a traditional camel breeding region that is nationally regarded as a prosperous herding hub. Bordering China, parts of the Soum are closer to Beijing than Ulaan Baatar, the capital city. The Oyu Tolgoi mine is in the western portion of the soum; the community claims this zone is the principal dust source in the region [15,80]. 

### 2.3. Dust Traps

**Figure 5:**
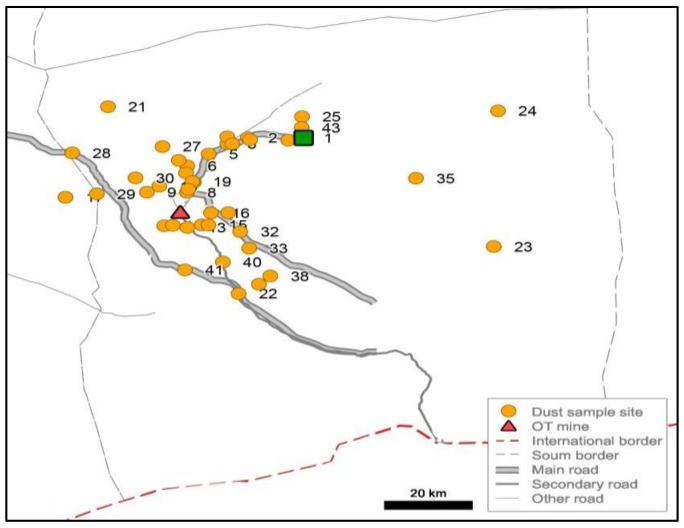
Dust trap placement in Khanbogd Soum.

**Figure 6:**
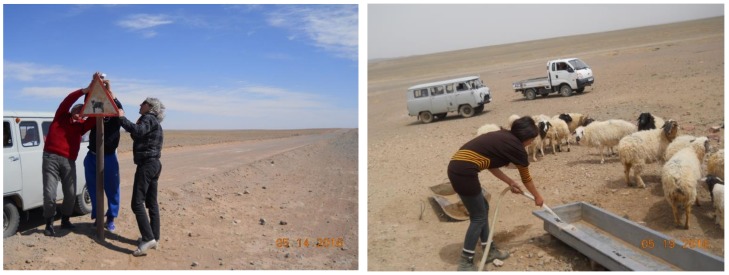
Dust trap placement on unpaved road between mine and town (**left**); steppe landscape in Khanbogd district (**right**). Photos by author.

To provide a local-scale assessment of dust, 43 dust traps were placed throughout the soum in May 2016 (Figure 5). The wide spread of sites was essential to identify aerosol dust deposition rates across the soum to determine areas of concentration. Each dust trap box (Figure 6) was of identical cardboard construction with dimensions of 100 × 60 × 30 mm and contained one ultra-fine filter paper (to retain micro-particles) and one absorbent sponge to retain larger particles. The traps were placed at 1.5 to 2 m height, with open tops to catch ambient dust in the air rather than ground-level saltating particles. In this environment, the lack of built structure meant that the trap height could not be kept precisely constant. 

Figure 6. Dust trap design with filter paper on bottom and a 15 mm thick dry sponge (yellow) filled the box. The sponge and box sides limited dust escaping from the trap. As an initial study deposition rate rather than grain size was assessed.

Dust traps were placed in the vicinity of the Oyu Tolgoi mine, including on the one main, unpaved road, at herder camps, villages, open countryside, water sources, and in the Soum centre (Figure 6). In this manner, daily airborne dust deposition rates were established throughout the district, enabling the identification of dust deposition configurations at a wide spatial scale. Because of the large sample area and the number of sites dust trap placement and collection occurred on different dates. This infers that environmental conditions may vary by monitoring dates and wind and rain conditions. The dust trap data provide a direct measure of deposition, though site comparison may fluctuate. MODIS satellite data was used to measure aerosol optical depth (AOD) to determine recent (2002–2016) dust history in the soum.

## 3. Results

**Figure 7:**
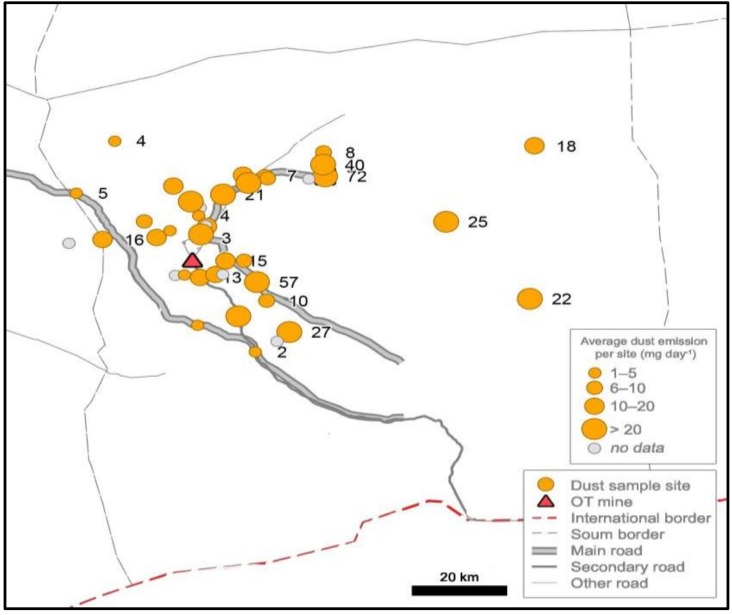
Distribution of dust emissions (mg day^−1^) across the survey sites in Khanbogd Soum. Larger circle denotes higher dust concentration.

Dust concentration varied considerably across the study area (Table 3; Figure 7). In May 2016, the highest deposition rates were found on the unpaved road from the mine to the town, at the most distant site (75 km from the mine), and near the town. Other sites on the unpaved road had significantly less dust, recording a fraction (1/4 to 1/7) of the amount. Though near the mine site, several traps had low dust deposition rates with traps that were 250 m from the unpaved road and near the paved road sections had the least dust. The old airport, village centres, and near the paved coal road had low volumes. Other factors, including weather, wind, topography, and natural setting, appear to be dust determinants, as well as nearness to commonly identify dust sites, which include the mine license area, unpaved roads, livestock concentrations, or the former airport zone.

- Findings along unpaved sections of the Khanbogd road were mixed with low to high volumes recorded.
- Nomgon, the village furthest from the mine (75 km), had more dust than the nearer villages Javalant (16 km) or Gavaluut (20 km).
- Some sites near OT were relatively low whilst locations east of the soum centre had higher concentration.
- Results and observation suggests that the Khanbogd road has limited dust impact beyond the immediate (to 250 m) vicinity.
- Initial source of the dust was not investigated, but it is likely that several other potential dust sources exist beyond mining and related infrastructure: railway construction, coal trucks, herder vehicles, unpaved roads unrelated to OT, quarries and dirt pits, dry riverbeds, areas of livestock concentration, and herder vehicle tracks.

**Figure 8:**
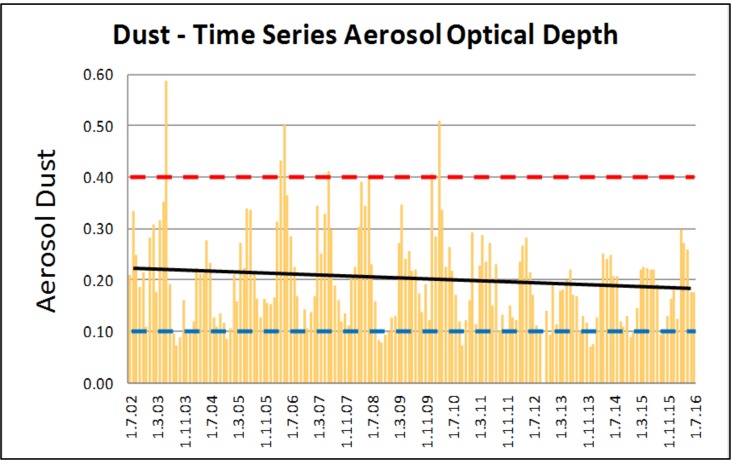
MODIS remotely sensed aerosol dust assessment in Khanbogd Soum programme [103]. A value of 0.1 = very clear—blue dotted line; 0.4 = very dusty—red dotted line. Black line represents aerosol dust trend from July 2002 to July 2016 showing slight decreasing trend in soum-wide dust over time (*r*^2^ = 0.033).

Assessment of potential dust levels over time, examined through aerosol optical depth analysis, shows a high variability in the 2000s. This may be associated with development and construction of the Oyu Tolgoi mine, the Tavan Tolgoi coal mine site north of the soum, and coal transport on unpaved roads (Figure 8). The Soum had elevated dust levels in 2005–2010, the lower levels from 2011 to 2016. There were variations between months and years with spring experiencing a raised dust level, probably due to the wind regime. Autumn had decreased dust. On NASA’s scale (0.1 clear conditions, 0.4 high dust) 2011–2016 dust concentration was satisfactory, fluctuating between 0.1 (good) and 0.3 (poor) dust conditions [102]. Indications are that dust is not a currently a major source of environmental disturbance.

Primary investigation focused on amount and distribution of dust. In arid environments residents grow up in dusty conditions, thus it is difficult to assess the ‘additionality’, or the source of the dust^34^. An example during the fieldwork is that OT’s Khanbumbat airport was temporarily closed due to dust storms on 22 May 2016. This reflects landscape-wide events and the fact that large-scale dust events occur in the desert environment; it is not possible to clearly identify or separate dust sources. When meeting with the director of the regional Khanbogd Hospital, the director stated unequivocally that there were no hospital admissions for dust-related symptoms in 2015–2016.

## 4. Discussion

Central and Inner Asia is a major dust source and transport region with potential significant impact on human populations. Covering a vast area, the region poses several dust-related challenges in identifying the source, particulate composition, natural vs. human causes, and community impact. A major factor and social concern is dust impact on health and its magnification by mining projects [75]. Our focused investigation on the Oyu Tolgoi mega-mine in Mongolia found that at present, there is little evidence of OT-sourced severe dust impact. Monitoring of particulate matter (i.e., PM_10_) in the mining area and the greater community identifies variable dust concentration with no clear signal indicating mining-driven dust impact or present exacerbation. This is comparable to data presented by Oyu Tolgoi (see ot.mn/our-operations for details). Time analysis reflects prior elevated dust levels (2005–2010) over the study area, whilst 2011 to 2016 show dust levels that are acceptable by UN standards. 

The greater region shows a number of evolving dust sources from both natural desert dynamics and its unusual recent political history, including emissions resulting from the Soviet-instigated Virgin Lands, the drying of the Aral Sea, and China’s ‘Conquer the West’ agricultural development programmes. Today large-scale mining, regional land cover change as a result of human activity, desertification, and climate processes further contribute to dust emissions. Interestingly, the study notes a declining trend in dust deposition in the region. This may be counterintuitive to an increase in identified dust sources and calls for further investigation through remote sensing and on-the-ground studies. The significance of Central and Inner Asian-sourced dust is a great concern for Beijing, Korea, and Japan where deposition occurs and is now monitored in the western United States.

Taking one focus region, the southern Mongolian Gobi Desert, provided a topical case study of dust in the human environment. Framed by mega-mining, community complaints to the national government, and international agencies highlighted the perceived health-related impact of mining-generated dust. Resulting global attention [94,104] provided the research context and stressed the value of on-the-ground investigation. The community expectation was that highest the dust concentration would be found at the mine perimeter, and decrease with distance from the mine. In fact, the documented random pattern suggests that natural factors, such as wind and geography, and human processes—vehicle transport on unpaved roads and dirt tracks, town location, and livestock grazing patterns—affect dust and can lead to levels higher than in the mine vicinity. Key points are that the desert location is an endemic dusty area, there was not an intensification or concentration of particulates nearest the mine site and that aerosol optical analysis found the soum-wide assessment of dust to be of reasonable quality for human health when measured in 2016. Comparatively high concentration at sites far afield from the mine (75 km), including beyond a range of hills that may inhibit dust transport, make the point that the mine is one of several dust sources. A detailed study focused on particulate chemical composition that matches the sources within the mine site and outside the mine license area would be needed to trace dust provenance in the local community, which experiences dust generated elsewhere in the Gobi or brought by wind and weather patterns. 

The findings are limited by the methodology; more in-depth study (minerology, grain size, biological matter, etc.) is required to determine health risk. Health testing, including spirometry, cardiovascular, and pulmonary tests and tissue sampling of livestock can more clearly identify exposure to dust in humans. This is important knowledge as mining, railways, and road construction are set to expand greatly in the Central Asian region through China’s One Belt, One Road $1 trillion infrastructure programme [105], Kazakhstan’s ‘Bright Path’ development plan [106], and ongoing resource extraction, e.g., in Kyrgyzstan, Mongolia and China. Whilst government monitoring of mining dust emissions is not strong (limited technical ability, cost, rent seeking, corruption, weak legal oversight), it is imperative for continued research to identify dust parameters in the region. Past history of dust’s deleterious effects in the region, including 2008’s 52 dust storm-related deaths in Mongolia [107] and toxic dust in the Aral Sea region ([108]; see also [4,5,6]), make further investigation into dust impact on health significant in Central and Inner Asia. 

## 5. Conclusions

Inner Asian drylands are known as a major global dust source that are driven by environmental factors and increased anthropogenic causes (new mining, development, expanded agriculture). In the region, the number of identified dust storms has been decreasing, whilst the volume of dust is uncertain. Investigation at one site, the $12 billion Oyu Tolgoi mine, found that contrary to local expectation, current dust concentration was dispersed in the community rather than intensified by the mine site. This suggests that mining is not significantly affecting the rates of human exposure to dust in the field site. In part, this reflects the high international mining standards used at Oyu Tolgoi and enforced state mining regulations; much regional mining is sub-standard and aggravates degradation.

This paper stresses the awareness of dust in greater Central Asia and identifies concern about related implications on health. Whilst documenting direct dust effect on humans was beyond the scope of the investigation, the works suggests that further study could examine impact through medical testing, chemical analysis of particulate, longer-term field study and detailed concentration, and composition evaluation. For example, traditional pastoral livelihoods in Asia offer the opportunity to test livestock as a proxy for human exposure to dust. Future human well-being in the region will require more thorough information on dust emissions in the changing environment. 

## Figures and Tables

**Table 1:** Central Asian dust framework.

Dust Sources	Causes-Physical	Causes-Human	Health Implications
Kyzyk Kum	Storms	Agriculture	Respiratory ailments
Kara Kum	- frequency	- irrigation	Lung disease
Taklamakan/Tarim	- intensity	- Virgin Lands programme	Cancer
Gobi (*including Badain Jaran, Tengger, Hexi, Junggar, Mu Us etc*.)	Climate change	- abandoned crops	Pneumonia
	Dried Aral Sea	Livestock grazing	Digestive ingestion
	Drought	Salinity	- dust, salt
Aral Kum	Wind	Pollution, contaminants	Eye, throat ailments
Moiyn Kum	Seasonality	Degradation	Malaria zones
Betpak Dash	Reduced vegetation/forest cover	Ground water depletion	Conjunctivitis
Pre-Balkash	Dessication of lakes	Off-road tracks	Menningitis
Turan Plain	Reduced soil moisture	Development, infrastructure	Cardovascular

**Table 2:** Literature on mining, dust and health in Mongolia.

Article	Topic	Author
Correlation between dust events in Mongolia and surface wind and precipitation.	Dusty days, strong winds and precipitatioin	Amgalan et al. 2017 [95]
Silica Dust from the enrich mining plant … origin in livestock	Mining dust impact on livestock	Tsetsegmaa 2004 [96]
Occupational lung diseases and the mining industry in Mongolia	Mining dust impact on humans	Lkhasuren et al. 2007 [97]
Environmental Review of Umnugobi Province and Negative Influence of Mining Industry to Livestock Health	Mining impact on livestock health	Orgil et al. 2011 [98]
Southern Gobi regional environmental assessment	Dust in the environment	World Bank 2010 [99]
Impact of the environment on health in Mongolia	Natural dust, mining-related dust	Jadambaa et al. [65]
Bringing health impact assessment to the Mongolian resource sector	Mining dust impact on human health	Byambaa et al. 2014 [100]
Dusty roads and disconnection:perceptions of dust from unpaved mining roads in Mongolia’s South gobi province	Perceived dust impact from Oyu tolgoi mine	Jackson 2015 [80]

**Table 3:** Dust deposition (mg day^−1^) by dust trap sites. Dust site locations by number are in Figure 7. *n.s.* = signifies no sample was collected. Reasons include trap eaten by a camel, birds withdrew sponge from trap, trap destroyed by weather and trap missing.

Dust Site	GPS N	GPS E	Depostion Rate per Day
1	43.20	107.19	0.072
2	43.20	107.03	0.007
3	43.18	106.98	0.001
4	43.20	106.98	0.017
5	43.16	106.92	0.021
6	43.13	106.86	*n.s.*
7	43.11	106.86	0.004
8	43.06	106.86	0.003
9	43.06	106.75	0.015
10	43.08	106.78	0.002
11	42.97	106.80	*n.s.*
12	42.97	106.82	0.003
13	42.97	106.86	0.013
14	42.98	106.90	0.012
15	42.98	106.92	*n.s.*
16	43.01	106.93	0.015
17	43.05	106.52	*n.s.*
18	43.09	106.88	0.011
19	43.09	106.88	*n.s.*
20	43.19	107.04	0.009
21	43.28	106.64	0.004
22	42.80	107.01	0.002
23	42.92	107.73	0.022
24	43.27	107.74	0.018
25	43.25	107.19	0.008
26	43.19	107.15	*n.s.*
27	43.18	106.79	0.019
28	43.16	106.54	0.005
29	43.06	106.61	0.016
30	43.10	106.72	0.007
31	43.01	106.98	0.01
32	42.96	107.01	0.057
33	42.92	107.04	0.01
34	43.14	106.84	0.022
35	43.10	107.51	0.025
36	43.18	106.99	0.083
37	43.05	106.52	*n.s.*
38	42.84	107.10	0.027
39	42.82	107.06	*n.s.*
40	42.88	106.96	0.075
41	42.86	106.86	0.005
42	43.07	106.87	0.076
43	43.23	107.19	0.04
